# *In vivo* Importance of Homologous Recombination DNA Repair for Mouse Neural Stem and Progenitor Cells

**DOI:** 10.1371/journal.pone.0037194

**Published:** 2012-05-29

**Authors:** Laure Rousseau, Olivier Etienne, Telma Roque, Chantal Desmaze, Céline Haton, Marc-André Mouthon, Jacqueline Bernardino-Sgherri, Jeroen Essers, Roland Kanaar, François D. Boussin

**Affiliations:** 1 Laboratoire de Radiopathologie, SCSR, iRCM, DSV, CEA, Fontenay-aux-Roses, France; 2 U967, INSERM, Fontenay-aux-Roses, France; 3 UMR 967, Université Paris Diderot, Sorbonne Paris Cité, Fontenay-aux-Roses, France; 4 UMR 967, Université Paris Sud, Fontenay-aux-Roses, France; 5 Laboratoire de Gamétogenèse, Apoptose et Génotoxicité, SCSR, iRCM, DSV, CEA, Fontenay-aux-Roses, France; 6 Department of Cell Biology & Genetics, Cancer Genomics Center, Erasmus MC, Rotterdam, The Netherlands; 7 Department of Radiation Oncology, Erasmus MC, Rotterdam, The Netherlands; 8 Department of Vascular Surgery, Erasmus MC, Rotterdam, The Netherlands; University Medical Center Hamburg-Eppendorf, Germany

## Abstract

We characterized the *in vivo* importance of the homologous recombination factor RAD54 for the developing mouse brain cortex in normal conditions or after ionizing radiation exposure. Contrary to numerous homologous recombination genes, *Rad54* disruption did not impact the cortical development without exogenous stress, but it dramatically enhanced the radiation sensitivity of neural stem and progenitor cells. This resulted in the death of all cells irradiated during S or G2, whereas the viability of cells irradiated in G1 or G0 was not affected by *Rad54* disruption. Apoptosis occurred after long arrests at intra-S and G2/M checkpoints. This concerned every type of neural stem and progenitor cells, showing that the importance of *Rad54* for radiation response was linked to the cell cycle phase at the time of irradiation and not to the differentiation state. In the developing brain, RAD54-dependent homologous recombination appeared absolutely required for the repair of damages induced by ionizing radiation during S and G2 phases, but not for the repair of endogenous damages in normal conditions. Altogether our data support the existence of RAD54-dependent and -independent homologous recombination pathways.

## Introduction

During development of the mammalian brain, neural stem and progenitor cells (NSPC) proliferate, undergo differentiation and migrate in a precisely coordinated manner before they become mature cell types in the central nervous system. Among the NSPC of the developing cortex, radial glia cells (RGC) functions as neural stem cells and generate neurons directly or indirectly *via* intermediate progenitors (IPC) [1], [2]. Although RGC possess long radial processes extending from the ventricular surface to the basal lamina, their nuclei are localized in the ventricular zone (VZ). RGC expand via symmetric divisions and perform asymmetric divisions to produce another RGC and an IPC or a neuron [2], [3]. Newborn IPC migrate to a more basal zone called the subventricular zone (SVZ) where they divide symmetrically to give a pair of IPC or a pair of neurons [4]. Newborn neurons migrate along the cytoplasm of RGC through the intermediate zone (IZ) to reach the cortical plate (CP), their final destination at the basal lamina [2], [4], [5].

RGC move their nuclei along their apical–basal axis, a process termed interkinetic nuclear migration (INM). They perform their mitosis at the ventricular surface and their S phase at the basal part of the VZ [6], [7]. INM and the durations of the different cell cycle phases regulate neurogenesis, through modulation of exposure of RGC to neurogenic signals which form a gradient in the developing brain [8], [9], [10].

DNA double-strand breaks (DSB) constitute one of the most challenging types of DNA damage. They can induce cell death or oncogenic chromosomal rearrangements [11]. DSB can be caused by either exogenous or endogenous stress (such as stalled replication forks) [11]. Mutations in DSB sensor and repair genes, such as *Mre11*, *Nbs1*, *ATR* and *ATM* can directly impair brain development and lead to brain pathologies such as microcephaly or mental deficiency [12], [13]. DSB are also the most relevant lesion for the deleterious effects of ionizing radiation (IR) [14]. Consistently, NSPC are highly prone to p53-dependent apoptosis after IR exposure [15], [16], [17], [18].

Nonhomologous end-joining (NHEJ) and homologous recombination (HR) constitute the two main pathways to repair DSB in mammalian cells. NHEJ is the most common pathway in multicellular eukaryotes for the repair of two-ended DSBs [19]. It performs a direct ligation of the DNA ends. HR is a more accurate and versatile mechanism of DSB repair. It uses an undamaged homologous DNA template and can repair one-ended DSBs occurring at replication forks [20]. But it takes more time to complete than NHEJ [21]. The lack of RAD51 or BRCA2, core proteins of HR, is lethal before neural development [22], [23]. However, conditional knockout of *Brca2* and deficiencies in other genes that participates in HR, such as *Xrcc2* or *fanca* and *fancg* resulted in an increase in apoptosis of cortical NSPC in mouse embryos [24], [25], [26], [27].

RAD54 is an important actor of HR (for a review [28]). Briefly, it interacts directly with RAD51 [29] and stimulates its DNA exchange activity [30]. It promotes chromatin remodeling [31], RAD51 displacement from double strand DNA [32], binds Holliday junctions and drives their branch migration [33]. *Rad54−/−* mouse embryonic stem cells [34], and chicken DT40 cells [35] are defective for HR. Although disruptions of other genes involved in HR lead to embryonic lethality, adult *Rad54−/−* mice are viable and fertile [34]. In this study, we determined the *in vivo* importance of *Rad54* for the developing mouse brain in normal conditions or after *in utero* IR exposure. Our results showed that *Rad54* disruption had no effect on cortical development in normal condition, but was strictly required for the survival of both RGC and IPC irradiated in S or G2/M, supporting the existence of RAD54-dependent and -independent HR pathways in NSPC. Altogether our data showed that the importance of *Rad54* for DNA repair in neural cells depends on the phase of the cell cycle during which DNA damage occurred and not on their differentiation stages.

## Materials and Methods

### Mice experiments

Mice experiments were carried out in compliance with the European Communities Council Directive of November 24, 1986 (86/609/EEC) and were specifically approved by our institutional committee on animal welfare (CETEA-CEA DSV IdF). Pregnant C57/Bl6 *Rad54*−/− [34] and *WT* mice were irradiated (1 Gy or 2 Gy, 0.6 Gy/min) at 14.5 days of gestation (E14.5) with a ^137^Cs source (IBL637, CIS BIO International). Intraperitoneal injections of 5-ethynyl-2′deoxyuridine (EdU, 100 µL at 1 mg/mL; ref A10044, Life technologies) and 5-bromo-2′-deoxyuridine (BrdU, 200 µL at 5 mg/mL; ref B5002, Sigma) were performed at different time points before or after irradiation as specified in the text or in the figures. After sacrifices, embryonic heads were removed and fixed by immersion overnight at 4°C in 4% paraformaldehyde (PFA) or at −20°C in methanol. Tissue was processed for paraffin embedding using a Tissu-tek processor (VIP; Leica). For histological analysis, 5 µm coronal sections were then obtained with a microtome (RM 2125 RT; Leica) and mounted onto glass slides.

### Terminal deoxynucleotidyl transferase labeling (TUNEL)

After paraffin removal, coronal sections were processed for TUNEL histochemistry according to the manufacturer's instructions (*In situ* cell death detection kit; Roche). Briefly, slices were boiled in citrate solution (pH6) and then incubated with the TUNEL reaction mixture that contained TdT and fluorescein-dUTP for 1 h at 37°C. After washes, the labels incorporated at the damaged sites of the DNA were visualized by fluorescence microscopy.

### Immunofluorescence and EdU staining

Paraffin-embedded tissue sections were deparaffinized, boiled in citrate solution (pH6) and then incubated in phosphate buffered saline (PBS) supplemented with 7.5% goat serum and 7.5% fetal bovine serum for 1 h at room temperature. Slides were incubated overnight with various primary antibodies at 4°C. The primary antibodies used were anti-Pax6 (1∶200; mouse; ref MAB1260; R&D), anti-cleaved caspase-3 (1∶200; rabbit; ref 9661; Cell Signaling), anti-Tbr2 (1∶200; rabbit; ref ab23345; Abcam), anti-Tbr1 (1∶200; rabbit; ref ab31940; Abcam) anti-acetyl- and phospho-histone 3 (lys9/ser10) (1∶200; rabbit; ref 9711; Cell Signaling) and anti-BrdU (1∶300; mouse; ref RPN202; GE Healthcare). After three washes, sections were incubated with either, Alexa Fluor 594 or 488 (1∶400; Life Technologies). Nuclear staining was achieved by incubation with 4′,6-diamidino-2-phenylindole (DAPI) to quantify apoptosis induction by the detection of pyknotic nuclei. Slides were mounted under Fluoromount (Southern Biotechnologies Associates), and sections were examined under a fluorescence microscope (Olympus BX51) with a 20× objective in three channels (appearing red, green and blue in the figures) as separates files. These images were then stacked with Photoshop software (Adobe) and used for enumeration of labeled nuclei.

EdU detection was performed after boiling in citrate solution and before saturation with blocking solution. Slices were permeabilized with 0.5% triton® X-100 in PBS for 15 min. EdU staining was performed according to the manufacturer's protocol (Click-iT EdU Alexa 488 imaging kit; ref C10083; Life Technologies). Briefly, the slices, protected from light, were incubated for 30 min with EdU labeling mix (Click-iT™ reaction buffer, CuSO_4_, Alexa Fluor® 488 Azide and reaction buffer additive).

Slices were analyzed by the use of a standard sector of the dorsomedial cerebral wall [36]. This sector was 100 µm in its medial-lateral dimension and was divided into 18 bins of 10 µm in height in its radial dimension. The sector was aligned such as the first bin was at the ventricular surface, with its long axis parallel to the ventricular border (Fig. S1A). The labeled (EdU, BrdU, Pax6 and/or Tbr2) and/or pyknotic nuclei were enumerated in each bin. Three different cortical slices were systematically analyzed by embryo. The experiments were always reproduced at least three times in independent manner. Results were indicated as mean +/− Standard error of the mean (SEM).

Statistical analyses were conducted with Graphpad Prism (Version 5.0c) using two-way ANOVA and Bonferroni multiple comparison posthoc tests or Mann-Whitney test, with significance measured as * p<0.05, ** p<0.01 and ***p<0.001.

## Results

### Disruption of *Rad54* does not significantly impair the cortical development

**Figure 1:**
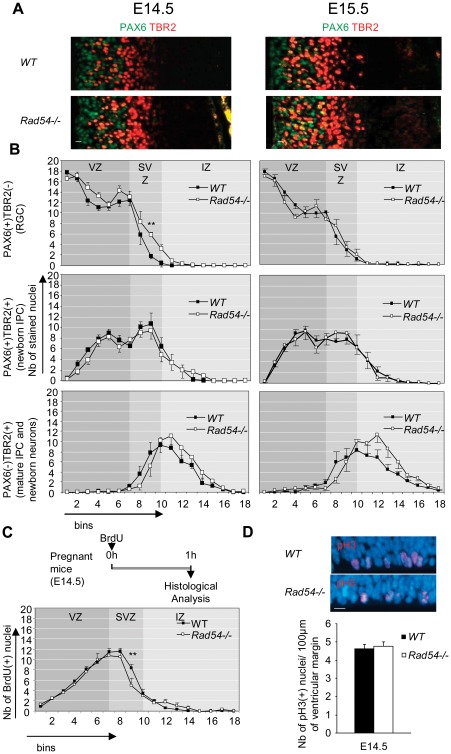
Disruption of *Rad54* has no effect on mouse cortical development. **A** Coronal section of the cerebral hemisphere of E14.5 (left) and E15.5 (right) *WT* (top) and *Rad54−/−* (bottom) embryos immunostained with PAX6 (green) and TBR2 (red). Ventricles are on the left of each section. Scale bars, 10 µm. **B** Number of PAX6(+)TBR2(−) (top), PAX6(+)TBR2(+) (middle) and PAX6(−)TBR2(+) (bottom) nuclei per bin at E14.5 (left) and E15.5 (right). Mean values ± SEM were calculated from *WT* (black squares) and *Rad54−/−* (open squares) embryos from at least three distinct litters for each genotype. **C** Top: scheme of experimental design. Bottom: Number of BrdU(+) nuclei per bin at E14.5, 1 h following a single injection of BrdU. Mean values ± SEM were calculated from *WT* (black squares) and *Rad54−/−* (open squares) embryos from at least three distinct litters for each genotype. **D** Top: Ventricle margins of hemispheres of E14.5 *WT* (left) and *Rad54−/−*(right) embryos stained by dapi (blue) and immunostained for pH3 (red). Scale bars, 10 µm. Bottom: Number of pH3(+) nuclei/100 µm of ventricular margin at E14.5. Mean values ± SEM were calculated from *WT* (black) and *Rad54−/−* (white) embryos from at least three distinct litters for each genotype.

Deficiency in DSB repair has been shown to impair brain development (For review [13]), we have thus investigated the consequences of *Rad54* disruption for the cortical development. We detected only rare apoptotic cells in the cortex of *Rad54−/−* and *WT* mouse embryos based on pyknotic nuclei, immunodetection of cleaved-caspase 3 and TUNEL assay (Fig. S1A). We then analyzed the expression of, PAX6 and TBR2 [37], [38], two nuclear markers allowing the identification of the different populations of NSPC at E14.5 and E15.5 (Fig. 1A) in a standard coronal sector of the dorsomedial cerebral wall (see the Materials and Methods and Fig. S1A). As shown in Fig. 1B, RGC (PAX6(+)TBR2(−) nuclei), newborn IPC migrating through the VZ and IPC of the SVZ (PAX6(+)TBR2(+) nuclei), mature IPC and the very newborn neurons (PAX6(−)TBR2(+) nuclei) were found in similar numbers and distributions in *Rad54* −/− and *WT* cortices. Moreover, the pattern of expression in the IZ and the CP of TBR1, a marker of postmitotic neurons [37] was similar in the two types of animals (Fig. S1C).

Since HR predominantly acts in S phase [39], we then investigated S phase cells by performing a pulse of BrdU, an analog of thymidine, and analyzed the number and localization of labeled nuclei in cortical slices after 1 h of BrdU incorporation. The number and the repartition of the BrdU(+) nuclei were similar in *WT* and *Rad54*−/− brains (Fig. 1C). Finally, we also found similar numbers of nuclei expressing phospho-histoneH3 (pH3), a marker of mitotic cells [40], at the ventricular surface in *WT* and *Rad54* −/− brains suggesting a similar rate of RGC mitoses (Fig. 1D).

Thus, we showed that *Rad54* disruption does not significantly impair the mouse cortical development, contrary to other genes involved in HR [24], [25], therefore extending prior works showing that Rad54 was not required for normal mouse development [34].

### Moderate increase in radiation-induced apoptosis of *Rad54−/−* NSPC within 8 h post-irradiation (PI)

**Figure 2:**
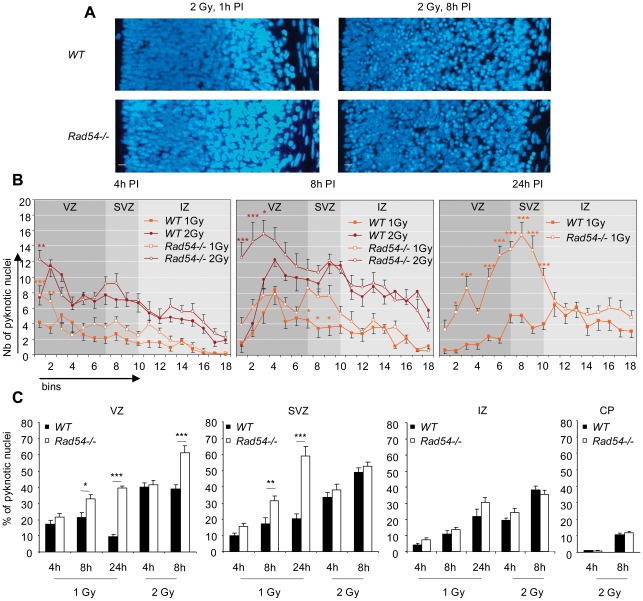
Radiation exposure induces apoptosis in developing cortex which is increased by *Rad54* disruption in NSPC. **A** Coronal section of the cerebral hemisphere of E14.5 *WT* (top) and *Rad54−/−* (bottom) embryos stained by dapi, 1 h or 8 h after a 2 Gy radiation exposure. Ventricles are on the left of each section. Scale bars, 10 µm. **B** Number of pyknotic nuclei per bin 4 h (left) 8 h (middle) or 24 h (right) after a 1 Gy (orange) or 2 Gy (brown) radiation exposure. Mean values ± SEM were calculated from *WT* (closed squares) and *Rad54−/−* (open squares) embryos from at least three distinct litters for each genotype. **C** Percentage of pyknotic nuclei in the VZ, the SVZ, the IZ and the CP at 4 h, 8 h or 24 h PI. Mean values ± SEM were calculated from *WT* (black) and *Rad54−/−* (white) embryos from at least three distinct litters for each genotype.

We then assessed the importance of *Rad54* for the developing cortex after DNA damage inducted by *in utero* (E14.5) exposure to IR. *Rad54−/−* mice are hypersensitive to IR at E3.5 [41]. Here, we tested two doses: 2 Gy, lethal at birth for *Rad54−/−* but not for *WT* embryos, and 1 Gy, not lethal for both types of animals (Table S1). As shown in Fig. 2A–B, no apoptotic nuclei were found in cortical slices at 1 h PI, but they were detected in a dose-dependent manner at 4 h and 8 h PI in the two types of animals. As we previously reported [15], radiation-induced apoptosis principally concerned the nuclei present from the VZ to the IZ and only few neurons of the CP (Fig. 2C). We observed a moderate increase in radiation-induced apoptosis that became significant at 8 h PI in the VZ and the SVZ of *Rad54−/*− mice, although most of radiation-induced apoptosis still occurred independently of the *Rad54* status (Fig. 2B–C). After 2 Gy, the number of apoptotic nuclei decreased and normally shaped nuclei reappeared in the first bins near ventricles from 4 h to 8 h PI in *WT* controls (Fig. 2B). By contrast, apoptosis increased and no normally shaped nuclei reappeared near ventricles within the same period in *Rad54−/−* mice. Altogether these results suggest that *Rad54* disruption increased radiation-induced apoptosis and may impair cell cycle progression and/or INM of irradiated NSPC.

### DNA damage response of NSPC depends on the phase during which they have been irradiated

**Figure 3:**
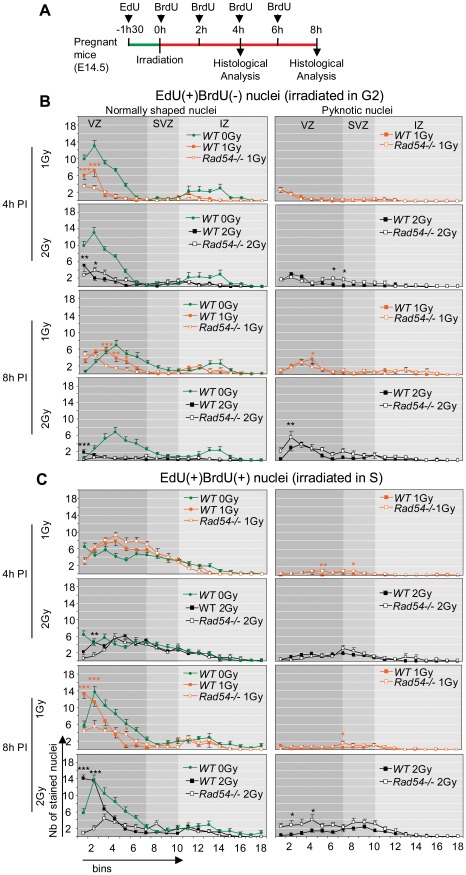
*Rad54* disruption lengthens G2/M arrest and delayed S-phase progression in S and G2 irradiated NSPC. **A** Scheme of the experimental design. Repeated BrdU injections were performed in order to insure perfect staining of all neural cells newly entering S phase during the 4 or 8 hours PI. **B and C** Number per bin of normally shaped (left) and pyknotic nuclei (right), from top to bottom, EdU(+)BrdU(−) (**B**) and EdU(+)BrdU(+) (**C**) nuclei 4 h (top) and 8 h (bottom) after a 1 Gy (orange), 2 Gy (black) or 0 Gy (green, control) radiation exposure. Mean values ± SEM were calculated from *WT* (closed squares) and *Rad54−/−* (open squares) embryos from at least three distinct litters for each genotype. Stars correspond to statistical analysis between *WT* and *Rad54−/−* curves.

It has been previously reported that NSPC spend approximately 4 h into S phase, 2 h into G2/M phases and 9.3 h into G1 phase in E14.5 mice embryos [42]. We labeled S-phase NSPC with EdU and BrdU, two analogues of thymidine, by successive intraperitoneal injections in pregnant mice (E14.5), to investigate the cell cycle progression of NSPC after irradiation, as previously described [18]. In a first set of experiments, EdU was injected 1.5 h before irradiation (1 or 2 Gy) and BrdU just after irradiation and then every two hours before the sacrifice of the animals at either 4 or 8 h PI (Fig. 3A). We have checked that neither EdU-, nor BrdU- incorporation changed the level of radiation-induced apoptosis in irradiated embryos (Fig. S2A) and that the sensitivity of EdU and BrdU detection did not allow the detection of their incorporation due to DNA repair, but only corresponded to DNA replication (Fig. S2B). Therefore, EdU incorporation revealed cells in S phase just before irradiation, whereas BrdU those after irradiation. The analysis of labeled nuclei in *WT* brains revealed marked differences in the consequences of irradiation depending on the phase during which neural cells had been irradiated. The followed protocol resulted in four types of differentially labeled nuclei: EdU(+)BrdU(−), EdU(+)BrdU(+), EdU(−)BrdU(+) and EdU(−)BrdU(−).

#### 1) EdU(+)BrdU(−) nuclei

They were in S phase before but not after irradiation. We have previously reported that S phase progression of irradiated neural progenitors is not blocked within 1 h PI [18] indicating thus that EdU(+)BrdU(−) nuclei were not irradiated in S phase. The mean duration of G2 and M phases is 2 h [38], [42], so the time lap of 1.5 h between EdU and BrdU, injections makes that EdU(+)BrdU(−) nuclei correspond to cells in G2 at the time of the first BrdU injection. Irradiation induced apoptosis of EdU(+)BrdU(−) nuclei within 8 h PI (Fig. 3B). As a consequence, we found a low number of surviving (normally shaped) EdU(+)BrdU(−) nuclei at 8 h PI in 2 Gy-irradiated *WT* mice (Fig. 3B). Four hours after 1 Gy, the number of living EdU(+)BrdU(−) nuclei was nearly two-times lower in *WT* mice compared to that of unirradiated controls (Fig. 3B, note that this concerned both RGC and IPC). But it was increased at 8 h PI (Fig. 3B), indicating that cells irradiated in G2 underwent mitosis within 4 h and 8 h PI. However, irradiated EdU(+)BrdU(−) nuclei had a more apical distribution than unirradiated controls at 8 h PI, evidencing a delayed apical to basal migration in irradiated brain (Fig. 3B). Altogether, these results confirmed our previous reports [18], that both RGC and IPC are highly radiosensitive during the G2 phase. They activated the G2/M checkpoint, which delayed the progression into mitosis of surviving cells from at least 2 h, but no more than 4 h, in 1 Gy-irradiated *WT* brains.

#### 2) EdU(+)BrdU(+) nuclei

**Figure 4:**
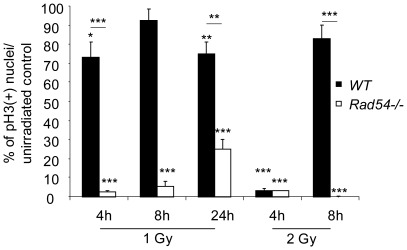
*Rad54* disruption delays mitotic cells reappearance after radiation exposure. Ratio between pH3(+) nuclei after irradiation and unirradiated control, expressed as percentage Mean values ± SEM were calculated from *WT* (black) and *Rad54−/−* (white) embryos from at least three distinct litters for each genotype. Stars above the histograms correspond to statistical comparison with unirradiated controls. Stars above lines correspond to statistical analysis between *WT* and *Rad54−/−* genotypes.

These nuclei correspond to cells irradiated in S phase. Their apoptosis increased within 8 h PI in *WT* brains (Fig. 3C). Eight hours after 1 or 2 Gy, most of surviving EdU(+)BrdU(+) nuclei were found in the three first bins near ventricles showing that they underwent basal to apical migration to reach the ventricular surface (Fig. 3C). Then, they performed mitosis as indicated by the increase in pH3-positive nuclei at the surface of ventricles at that time (Fig. 4). However, comparison with unirradiated controls clearly shows that radiation also significantly delayed interkinetic migration of these nuclei (Fig. 3C). Therefore *WT* cells that were irradiated during S phase activated intra-S checkpoints, which were successfully passed by most of them within 8 h PI in *WT* brains. Importantly, the arrival of living EdU(+)BrdU(+) nuclei at the surface of ventricles appears as the main cause of the decrease in the number of apoptotic nuclei observed in the first bins of *WT* brains between 4 h and 8 h PI.

#### 3) EdU(−)BrdU(+) nuclei

**Figure 5:**
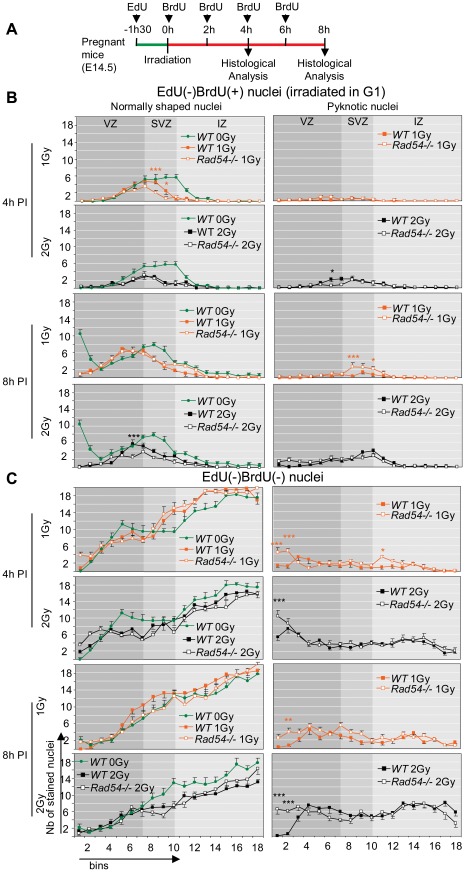
No interference of *Rad54* disruption with radiation response of G1 and postmitotic cells within 8 hPI. **A** Scheme of the experimental design. **B and C** Number per bin of normally shaped (left) and pyknotic nuclei (right), from top to bottom, EdU(−)BrdU(+) (**B**) and EdU(−)BrdU(−) (**C**) nuclei 4 h (top) and 8 h (bottom) after a 1 Gy (orange), 2 Gy (black) or 0 Gy (green, control) radiation exposure. Mean values ± SEM were calculated from *WT* (closed squares) and *Rad54−/−* (open squares) embryos from at least three distinct litters for each genotype. Stars correspond to statistical analysis between *WT* and *Rad54−/−* curves.

They correspond to cells irradiated in G1 that entered S phase after irradiation. Radiation decreased their number, induced their apoptosis in a dose-dependent manner and delayed their interkinetic nuclear migration as evidenced by the absence of EdU(−)BrdU(+) nuclei in the first bins near ventricle at 8 h PI contrary to unirradiated controls (Fig. 5B). Both apoptosis and delayed interkinetic migration suggest the activation of intra-S checkpoint activation in these cells (Fig. 5B).

#### 4) EdU(−)BrdU(−) nuclei

Most apoptotic nuclei at 4 h and 8 h PI were EdU(−)BrdU(−) as shown in Fig. 5C. These nuclei correspond to cells that were not in S phase from 1.5 h before to 4 h or 8 h after radiation and therefore to cells irradiated in G1 (RGC and IPC) or in G0 (postmitotic neurons). Apoptosis of EdU(−)BrdU(−) nuclei in the IZ clearly shows that migrating postmitotic neurons, contrary to neurons of the cortical plate, are sensitive to radiation (Fig. 5C). The mean duration of G1 of NSPC at E14.5 mice is 9.3 h [42]. Interestingly, the number of apoptotic EdU(−)BrdU(−) nuclei in the VZ and the SVZ largely exceeds the decrease in EdU(−)BrdU(+) nuclei observed at 8 h PI. Our results suggest therefore that most apoptotic EdU(−)BrdU(−) nuclei in the VZ and SVZ decreased the production of postmitotic neurons generated from RGC and IPC, as previously proposed [18].

### *Rad54* disruption lengthens G2/M arrest and delayed S-phase progression in irradiated NSPC

We did not find any significant differences between the numbers and/or distribution of EdU- and/or BrdU-labeled nuclei within unirradiated *WT* and *Rad54−/−* cortical slices (Fig. S3), confirming absence of an essential function of *Rad54* for the progression of the cell cycle of NSPC in normal conditions. By contrast, *Rad54* disruption had a different impact on the DNA damage response of NSPC depending on the phase of the cell cycle during which they had been irradiated and the radiation dose.

After 2 Gy of IR the amount of induced DNA damage is of such a magnitude that most EdU(+)BrdU(−) nuclei in both *Rad54−/−* and *WT* mice died within 8 h PI (Fig. 5B). So, a contribution of *Rad54* towards the promotion of survival of cells irradiated in G2 was not detected in those conditions. However, after 1 Gy, the number of living EdU(+)BrdU(−) nuclei did not increase from 4 h to 8 h PI (Fig. 3B–C) in *Rad54−/−* mice contrary to *WT* controls. Consistently, quantification of pH3-positive nuclei at the surface of the ventricles showed, in *WT* brains, a dose-dependent decrease in the number of pH3-positive nuclei at 4 h PI, which returned to the control level at 8 h PI. By contrast, pH3(+) nuclei did not reappear (2 Gy), or reappeared with a slower rate (1 Gy) at 8 h PI in *Rad54*−/− brains (Fig. 4). This suggests that *Rad54* disruption lengthens the G2/M arrest from at least 6 h in 1 Gy-irradiated *Rad54−/−* cells, as compared to no more than 4 h in *WT* cells as shown above.

Contrary to cells irradiated in G2, *Rad54* disruption increased apoptosis of cells irradiated in S phase (EdU(+)BrdU(+) nuclei) within 8 h PI, but this increase is low and detected only at the highest dose (Fig. 3B–C). Moreover, even after 2 Gy, most of S-phase cell death occurred independently of the *Rad54* status. Importantly, *Rad54* disruption resulted in a decrease in EdU(+)BrdU(+) nuclei present in the first bins near ventricle at 8 h PI. They were two-times reduced compared to irradiated *WT* controls after 1 Gy, and nearly absent after 2 Gy. This suggests that *Rad54* disruption lengthened S-phase progression as a consequence of intra-S checkpoints activation delaying (1 Gy) or preventing (2 Gy) the transition from S to G2 and INM within 8 h PI.

### No interference of *Rad54* disruption with radiation response of G1 and postmitotic cells within 8 h PI

Except minor variations, *Rad54* disruption did not affect the number and distribution of EdU(−)BrdU(+) nuclei in cortical slices within 8 h PI (Fig. 5B). Similarly no major differences in EdU(−)BrdU(−) nuclei were observed between irradiated *Rad54−/−* and *WT* mice in SVZ and IZ and in the upper bins of the VZ (Fig. 5C). By contrast, the number of apoptotic EdU(−)BrdU(−) nuclei increased in the first bins near ventricles of irradiated *Rad54−/−* brains. The position of these nuclei suggests that they correspond to RGC that were already in G2 when EdU was injected, then irradiated either in late G2 or M phase, and dying thereafter at the border of the ventricles. Their presence in the first bins near ventricles at 8 h PI in *Rad54−/−* mice is thus likely the consequence of the decreased arrival of living nuclei at the ventricular surface due to the arrest of INM. Altogether *Rad54* disruption did not interfere with radiation response of G1 and postmitotic cells within 8 h PI.

### Total loss of *Rad54*−/− RGC and IPC irradiated during S-G2 phases and of their progeny at 24 h PI

We then investigated the consequences of irradiation at longer times after irradiation. *Rad54* disruption dramatically enhanced cell death at 24 h PI, which resulted in *Rad54*−/− brains that were too damaged to be analyzed after 2 Gy. Further experiments were thus limited to 1 Gy irradiation.

**Figure 6:**
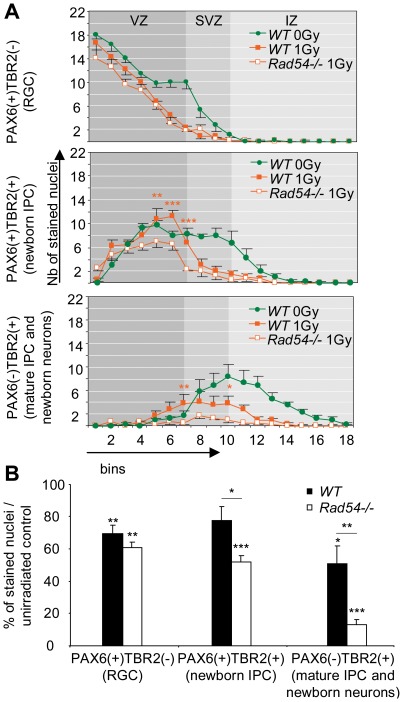
Radiation exposure induces massive apoptosis and G2/M arrest in NSPC. **A** Number of PAX6(+)TBR2(−) (top), PAX6(+)TBR2(+) (middle) and PAX6(−)TBR2(+) (bottom) nuclei per bin 4 h after a 1 Gy (orange) or 0 Gy (green, control) radiation exposure. Mean values ± SEM were calculated from *WT* (closed squares) and *Rad54−/−* (open squares) embryos from at least three distinct litters for each genotype. Stars correspond to statistical analysis between *WT* and *Rad54−/−* curve. **B** Ratio between total PAX6(+)TBR2(−) (left), PAX6(+)TBR2(+) (middle) and PAX6(−)TBR2(+) (right) nuclei and unirradiated control, expressed as percentage. Mean values ± SEM were calculated from *WT* (black) and *Rad54−/−* (white) embryos from at least three distinct litters for each genotype.

In *WT* brains, radiation-induced apoptosis remained at level similar to that at 8 h PI in the SVZ or increased in the IZ (Fig. 2C). Furthermore, immunophenotyping revealed that the number of mature IPC and newborn neurons cells decreased in these zones compared to unirradiated controls (Fig. 6A–B). By contrast, apoptosis decreased in the VZ from 8 to 24 h PI (Fig. 2B–C) and the composition in RGC and newborn IPC of bins 1 to 5 became almost identical to that of unirradiated controls, providing evidence for the ongoing reconstitution of the VZ after radiation-induced cell death (Fig. 6A).

*Rad54* disruption led to a significant increase in radiation-induced apoptosis that peaked in the SVZ in which it concerned 59±6% of nuclei as compared to 20±3% in *WT* brains (Fig. 2B–C). This was associated with a decrease in newborn IPC and the almost complete loss of mature IPC and very newborn neurons (Fig. 6A–B). But quite surprisingly, the numbers and the distributions of RGC in *Rad54−/−* embryos were identical to irradiated *WT* controls (Fig. 6A–B). Furthermore, as in *WT* brains, RGC and newborn IPC were in similar proportions to unirradiated controls in the first 4 bins near ventricles. Altogether, despite a massive apoptosis, the progressive reconstitution of the VZ after irradiation observed in *WT* brains also occurred in *Rad54*−/− mice at 24 h PI, consistently with our observation that the animals survived after 1 Gy-irradiation (Table S1).

**Figure 7:**
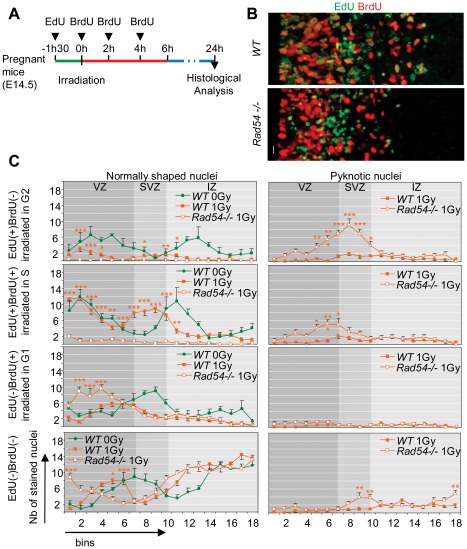
Consequences of 1 Gy radiation exposure 24 h PI for cells irradiated during different cell cycle phases. **A** Scheme of the experimental design. **B** Coronal sections of the cerebral hemisphere 24 h after 1 Gy radiation exposure and injected with EdU and BrdU like described in (A) from *WT* (top) and *Rad54−/−* (bottom) embryos stained for EdU (green) and BrdU (red). Ventricles are on the left of each section and the right margin is in the middle of IZ. Scale bars, 10 µm. **C** Number per bin of normal shaped (left) and pyknotic (right) of, from top to bottom, EdU(+)BrdU(−), EdU(+)BrdU(+), EdU(−)BrdU(+) and EdU(−)BrdU(−) nuclei 24 h after a 1 Gy (orange) or 0 Gy (green, control) radiation exposure. Mean values ± SEM were calculated from *WT* (closed squares) and *Rad54−/−* (open squares) embryos from at least three distinct litters for each genotype. Stars correspond to statistical analysis between *WT* and *Rad54−/−* curves.

We thus performed another set of labeling experiments in which we injected pregnant mice with EdU 1.5 h before, and BrdU just after radiation exposure (0 h), and at 2 h and 4 h PI with a dose allowing its incorporation for no more than 2 h in cycling neural progenitors (Fig. 7A). The animals were then sacrificed at 24 h PI. As in the preceding experiment, no significant differences in number and distribution of labeled cells were found in unirradiated *WT* and *Rad54*−/− cortical slices (Fig. S3).

As shown in Fig. 7B–C, radiation exposure led to important changes in the pattern of BrdU and/or EdU stained nuclei in *WT* brains. The number of EdU(+)BrdU(−) nuclei decreased much more than the other types of stained nuclei, confirming that NSPC are more radio-sensitive during G2. Moreover, the other categories of nuclei had a more apical distribution in irradiated brains, suggesting that radiation had induced a general delay in INM and neurogenesis. Interestingly, apoptosis detected in the SVZ and the IZ concerned mostly EdU(+)BrdU(−) or EdU(+)BrdU(+) nuclei, whereas apoptosis of EdU(−)BrdU(−) nuclei decreased compared to 8 h PI.

*Rad54* disruption resulted (Fig. 7C) in the almost complete loss of cells that were irradiated in G2 (EdU(+)BrdU(−) nuclei), and of those irradiated in S phase (EdU(+)BrdU(+) nuclei), whereas the majority of their *WT* counterparts survived. Consistently, the increase in apoptosis observed in *Rad54−/−* mice was related to the massive death of EdU(+)BrdU(−) and EdU(+)BrdU(+) cells occurring within 8 h and 24 h PI. Therefore *Rad54* is strictly required for the survival of NSPC irradiated during S or G2 and of their progeny, demonstrating the major role of HR for the repair of DNA damages during these phases.

Moreover, *Rad54* disruption led to important changes in the distribution of living EdU(−)BrdU(+) or EdU(−)BrdU(−) nuclei in the VZ compared to *WT* controls (Fig. 7B–C) and to a slight increase in apoptosis of EdU(−)BrdU(−) nuclei (limited to the SVZ), but not of EdU(−)BrdU(+) nuclei (Fig. 7C). Interestingly, as indicated above, RGC and newborn IPC were found in similar numbers and distributions in the first bins near ventricle in irradiated *WT* and *Rad54*−/− mice (Fig. 6A), but they were not irradiated in the same phase of the cell cycle in the two types mice (Fig. 6C). Thus, RGC irradiated in G1 (EdU(−)BrdU(+) or EdU(−)BrdU(−) nuclei) have compensated the massive death of RGC irradiated in S or G2 phases (EdU(−)BrdU(+) and EdU(−)BrdU(−) nuclei) to restore the pool of cycling progenitors in *Rad54*−/− mice. This sustains the hypothesis of a reorientation of these cells toward self-renewal rather than neurogenesis, that should be further investigated.

## Discussion

Setting up an efficient DNA damage response leading to the accurate repair or elimination of damaged cells is an important challenge for highly proliferative fetal stem cells, which are supposed to insure the fidelity of the transmission of their genomes to their progeny and supposed to preserve both self-renewal capacity and multipotency. Compared to NHEJ, HR is a more accurate method to repair DSB, and has a crucial importance for NSPC during brain development [43]. In this study, we have investigated the *in vivo* consequences for NSPC of the disruption of *Rad54*, an important actor of HR. Contrary to the inactivation of major HR factors, leading to early embryonic lethality [22], [23], *Rad54* disruption does not impair mouse viability [34] and we have shown here that it did not impact the cortical development in normal conditions. *Rad54−/−* mice are hypersensitive to IR at the embryonic, but not at the adult stage [41]. However, the relation between the cell cycle and radiation response was not investigated to date in these mice. Our study demonstrates that *Rad54* is absolutely required for long-term survival of both RGC and IPC irradiated during S and G2 phases. Disruption of *Rad54* resulted in their apoptosis occurring after the dramatic lengthening of intra-S and G2/M checkpoints compared to *WT* controls, in association with strong perturbations of INM, which has a major role in the cell fate determination [8], [9], [10], [44]. By contrast, *Rad54* disruption did not radio-sensitize post-mitotic neurons and NSPC in G1 phase. These results suggest that *Rad54* is crucial for the correct repair of DNA damage induced by IR in NSPC during the S and G2 phases, but not for the repair of those occurring in G1 or G0. During these phases, the DNA damage can be repaired by NHEJ [45]. So, the higher radiation sensitivity of *Rad54−/−* mice at embryonic stage could be explaining by a higher level of cell proliferation.

We have previously reported that *in vivo*, NSPC failed to exhibit a block at G1/S transition after *in utero* exposure to IR, leading to unaltered entry of neural progenitors into S phase [18]. Interestingly, we have observed that NSPC irradiated in G1, which entered S phase after irradiation died in a dose-dependent manner within 8 h PI. This suggests that these cells entered in S phase with unrepaired DNA damages resulting in the activation of intra-S checkpoints and subsequently apoptosis. Not only the level of apoptosis of these cells was not increased in *Rad54−/*− mice at 8 h PI, but a larger number of them were found alive in *Rad54*−/− compared to *WT* VZ at 24 h PI, indicating the lack of importance of *Rad54* for DNA repair in these cells after irradiation. Altogether we showed that DNA damage occurring in G1 in NSPC are not handled in S phase by HR, ruling out the hypothesis that this mechanism could insure the fidelity of DNA repair in NSPC entering S phase after irradiation because of the lack of a functional G1/S checkpoint [18].

The importance of HR for NSPC during brain development has been demonstrated by the detection of high levels of apoptosis in the developing cortex of conditional *Brca2* mutants [25] and of *Xrcc2*−/− embryos that survived early lethality [24]. By contrast, *Rad54* inactivation generates a very low increase (approximately 1 apoptotic cell/1000 cells) in apoptosis in the mouse developing cerebellum at postnatal day 1 [46] and here we show the absence of significant increase in apoptosis in the developing cortex of *Rad54*−/− mice compared to *WT* controls. *Rad54* inactivation does not totally abrogate HR [34]. Between 6% and 12% of HR activity persist in *Rad54−/−* cells [47], [48], as a possible consequence of the existence of paralogs with overlapping functions, such as *Rad54B* [49]. It has been thus hypothesized that the level of endogenous DNA damage might be low enough to be effectively handled by HR in *Rad54−/−* cells allowing the normal development of *Rad54−/−* mice. However, in contrast to the phenotypes we observed after DNA damage induced by irradiation, no differences in cell cycle progression or in the level of spontaneous apoptosis were detected between unirradiated *WT* and *Rad54−/−* embryos. This indicates that Rad54 disruption did not delay or prevent repair of spontaneous DNA damage and suggests thus that the reason why disruption of Rad54 does not impact normal development is not related to a low amount of spontaneous damage, but to the type of lesion with HR having no need for Rad54 to handle them. Rad54 would be thus required for HR to handle DNA damage induced by IR, but not endogenous damage, which is consistent with the existence of distinct subpathways of HR, either dependent and independent of Rad54 as recently demonstrated [50].

The present study constitutes the first *in vivo* analysis of the importance of HR for mammalian cells in function of the cell cycle. Previous data obtained in cell cultures [21], [51], [52], have pointed that the importance of HR for mammalian cells varies according to the cell cycle, contrary to NHEJ that can contribute to DSB repair during all the cell cycle [53]. The commonly accepted explanation is that during the S and G2 phases the presence of the sister chromatid provides the undamaged template that is needed to complete HR [54]. This is associated with a regulation of the levels of critical HR proteins, including *Rad54*, which increase from S to G2 phase (For review [53]). Importantly, we showed for the first time, that RAD54-dependent HR was strictly required for the survival of NSPC after DNA damage occurring during S and G2 phases. Whether this concerns every type of cells or is limited to NSPC remained to be determined. However, we showed that this need of *Rad54* irradiated in S and G2 concerned RGC as well as IPC, showing that the important factor here is the cell cycle phase at the time of radiation exposure and not the differentiation stage of NSPC.

## Supporting Information

Figure S1 **Disruption of** ***Rad54*** **has no effect on mouse cortical development.** **A** Coronal section of the cerebral hemisphere *Rad54−/−* embryos stained with dapi (blue, top), TUNEL (green, middle) and cleaved caspase 3 (red, bottom) 8 h after a 0 Gy (left, control) or 2 Gy (right) radiation exposure. Ventricles are on the left of each section. Scale bars, 10 µm. **B** Top: Coronal section of E14.5 embryo stained with dapi. Red rectangle corresponds to the enlarged section shown below, which displays a coronal section of the cerebral hemisphere of E14.5 *WT* embryos stained with dapi and with an example one standard sector. This sector is 100 µm in its medial-lateral dimension and was divided into 18 bins of 10 µm in height in its radial dimension. The bins are number on the side of the sector and the VZ, SVZ, IZ and CP are represented. Ventricle is on the left of the section. **C** Coronal section of the cerebral hemisphere of E14.5 (left) and E15.5 (right) *WT* (top) and *Rad54−/−* (bottom) embryos immunostained with PAX6 (green) and TBR1 (red). Ventricles are on the left of each section. Scale bars, 10 µm.
(PDF)

Figure S2 **EdU and BrdU incorporations correspond to DNA replication and have no consequence on the induction of apoptosis.** **A** Number of pyknotic nuclei per bin 8 h after a 2 Gy radiation exposure with (open squares) or without (plain squares) injection of EdU and BrdU. Mean values ± SEM were calculated from *WT* (left) and *Rad54−/−* (right) embryos from at least three distinct litters for each genotype. All statistical analyses were performed as described in the Material and Methods. **B** Number of BrdU(+) nuclei per bin 1 h after a 0 Gy (plain squares) or 2 Gy (open squares) radiation exposure just followed by one injection of BrdU. Note that the distribution of BrdU(+) nuclei remained unaffected by irradiation indicating that BrdU incorporation is directly related to DNA replication in S phase. This indicates that the technique of BrdU detection used in these experiments was not sufficiently sensitive to detect DNA synthesis associated with DNA repair. Mean values ± SEM were calculated from *WT* embryos from at least three distinct litters for each group. All statistical analyses were performed as described in the Material and Methods.
(PDF)

Figure S3 **Disruption of** ***Rad54*** **has no effect on the progression of the cell cycle of NSPC in normal conditions.** **A** Scheme of the three experimental designs with analyses at 4 h, 8 h, and 24 h after the first injection of BrdU. **B** Coronal sections of the cerebral hemisphere of *WT* (top) and *Rad54−/−* (bottom) embryos coming from the protocol described in (A) at 4 h (left) or 24 h (right) after first injection of BrdU from embryos. The sections were stained for EdU (green) and BrdU (red). Ventricles are on the left of each section. Scale bars, 10 µm. **C** Number per bin of, from top to bottom, EdU(+)BrdU(−), EdU(+)BrdU(+), EdU(−)BrdU(+) and EdU(−)BrdU(−) nuclei of embryos coming from the protocols described in (A) with analyses 4 h (left), 8 h (middle) and 24 h (right) after the first injection of BrdU. Mean values ± SEM were calculated from *WT* (plain squares) and *Rad54−/−* (open squares) embryos from at least three distinct litters for each genotype. All statistical analyses were performed as described in the Material and Methods.
(PDF)

Table S1 **Survival after birth of** ***in utero*** **irradiated embryos at E14.5.** In each group, half were killed at 3.5 months, totally healthy. These others were alive up to 8 months.
(DOC)
