# Supplementary figures and images for: In vivo Importance of Homologous Recombination DNA Repair for Mouse Neural Stem and Progenitor Cells
Source: PLoS One. 2012 May 29;7(5):e37194. doi: 10.1371/journal.pone.0037194 (PMC3362579; doi:10.1371/journal.pone.0037194)

A

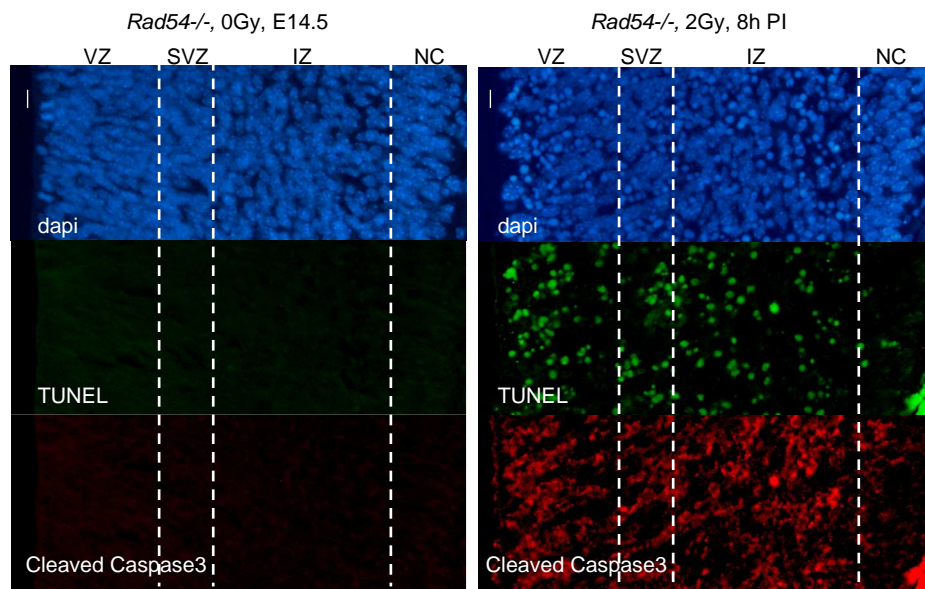

B

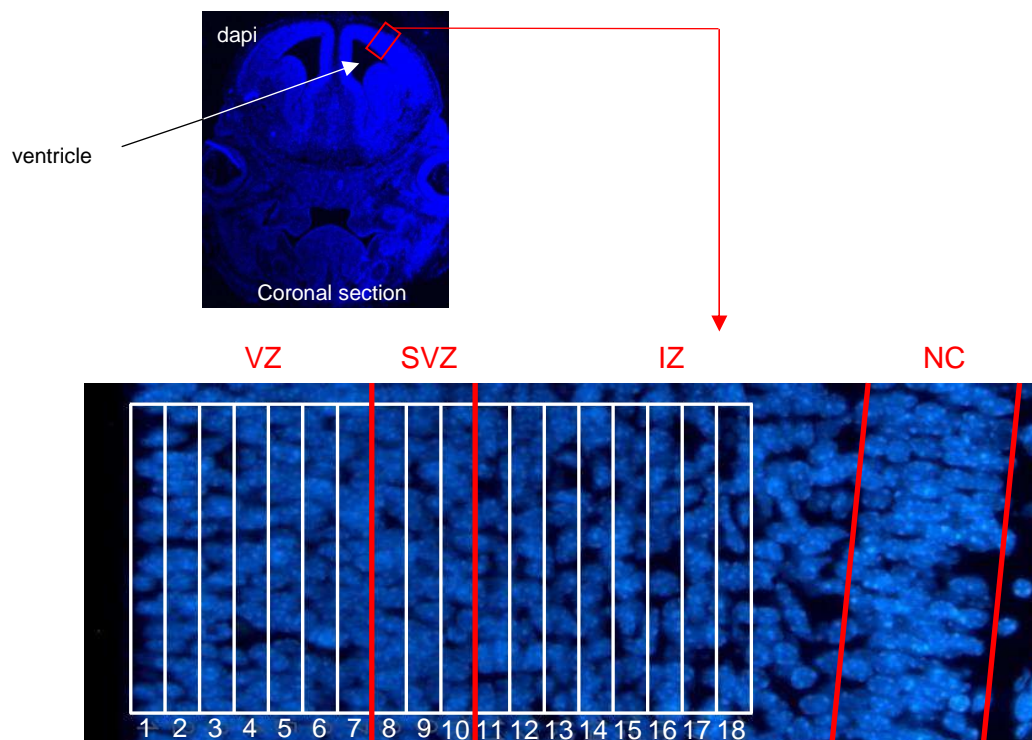

C

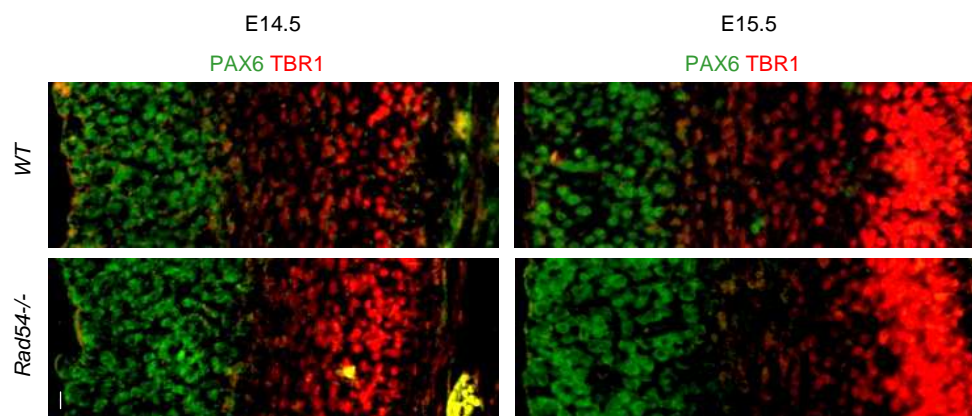

Fig.S1

Supplement: Figure S1 — Disruption of Rad54 has no effect on mouse cortical development. A Coronal section of the cerebral hemisphere Rad54−/− embryos stained with dapi (blue, top), TUNEL (green, middle) and cleaved caspase 3 (red, bottom) 8 h after a 0 Gy (left, control) or 2 Gy (right) radiation exposure. Ventricles are on the left of each section. Scale bars, 10 µm. B Top: Coronal section of E14.5 embryo stained with dapi. Red rectangle corresponds to the enlarged section shown below, which displays a coronal section of the cerebral hemisphere of E14.5 WT embryos stained with dapi and with an example one standard sector. This sector is 100 µm in its medial-lateral dimension and was divided into 18 bins of 10 µm in height in its radial dimension. The bins are number on the side of the sector and the VZ, SVZ, IZ and CP are represented. Ventricle is on the left of the section. C Coronal section of the cerebral hemisphere of E14.5 (left) and E15.5 (right) WT (top) and Rad54−/− (bottom) embryos immunostained with PAX6 (green) and TBR1 (red). Ventricles are on the left of each section. Scale bars, 10 µm. (PDF) [file pone.0037194.s001.pdf]

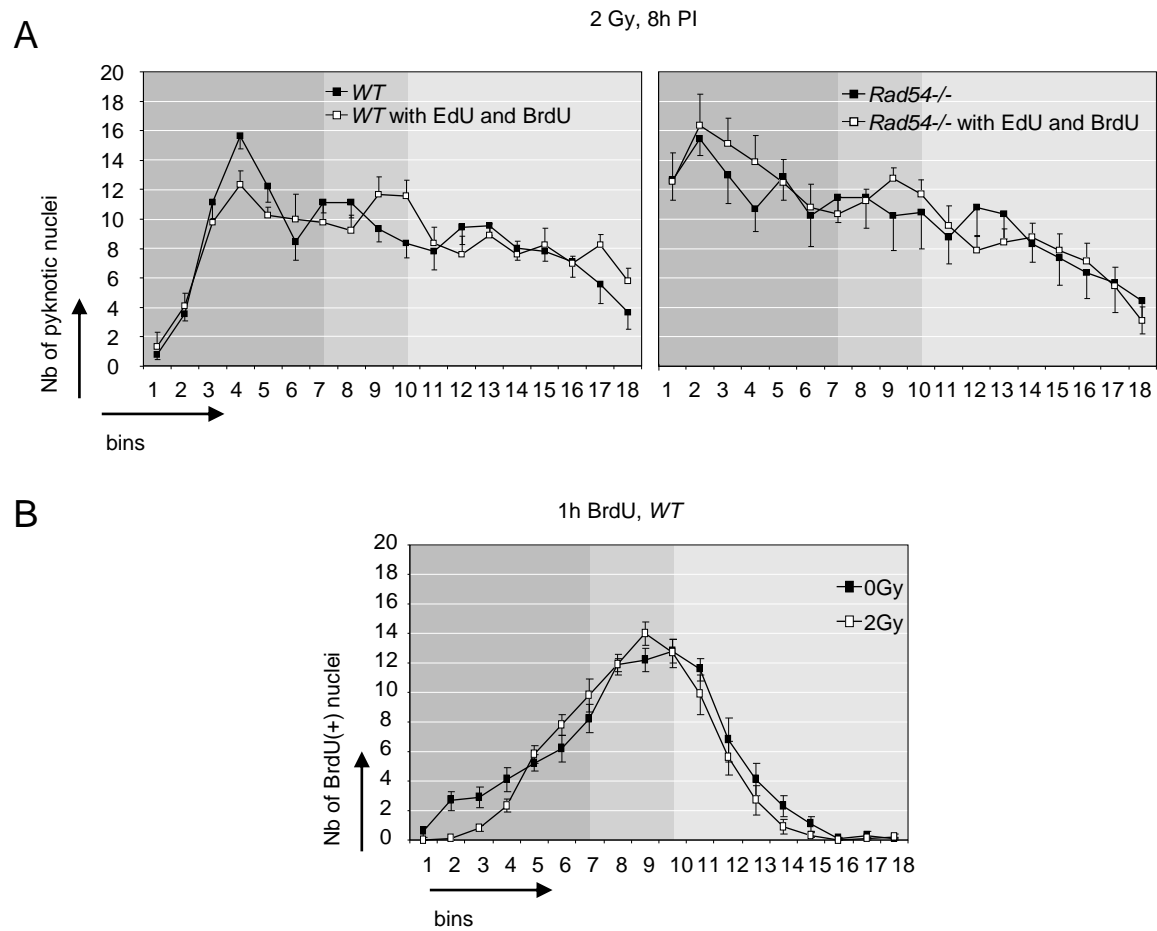

Fig.S2

Supplement: Figure S2 — EdU and BrdU incorporations correspond to DNA replication and have no consequence on the induction of apoptosis. A Number of pyknotic nuclei per bin 8 h after a 2 Gy radiation exposure with (open squares) or without (plain squares) injection of EdU and BrdU. Mean values ± SEM were calculated from WT (left) and Rad54−/− (right) embryos from at least three distinct litters for each genotype. All statistical analyses were performed as described in the Material and Methods. B Number of BrdU(+) nuclei per bin 1 h after a 0 Gy (plain squares) or 2 Gy (open squares) radiation exposure just followed by one injection of BrdU. Note that the distribution of BrdU(+) nuclei remained unaffected by irradiation indicating that BrdU incorporation is directly related to DNA replication in S phase. This indicates that the technique of BrdU detection used in these experiments was not sufficiently sensitive to detect DNA synthesis associated with DNA repair. Mean values ± SEM were calculated from WT embryos from at least three distinct litters for each group. All statistical analyses were performed as described in the Material and Methods. (PDF) [file pone.0037194.s002.pdf]

A

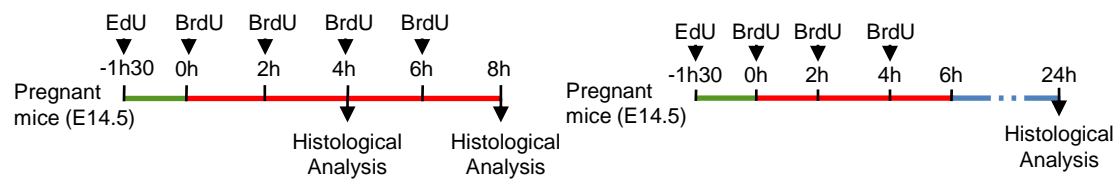

B

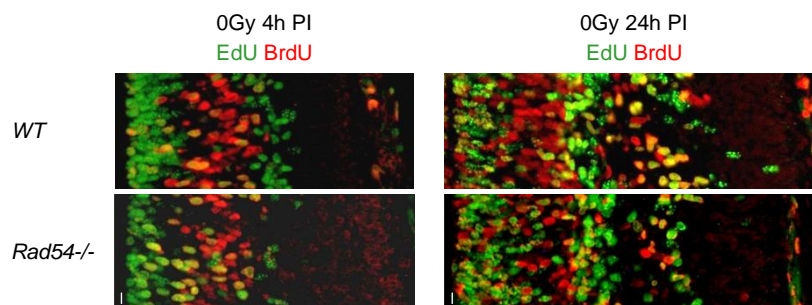

C

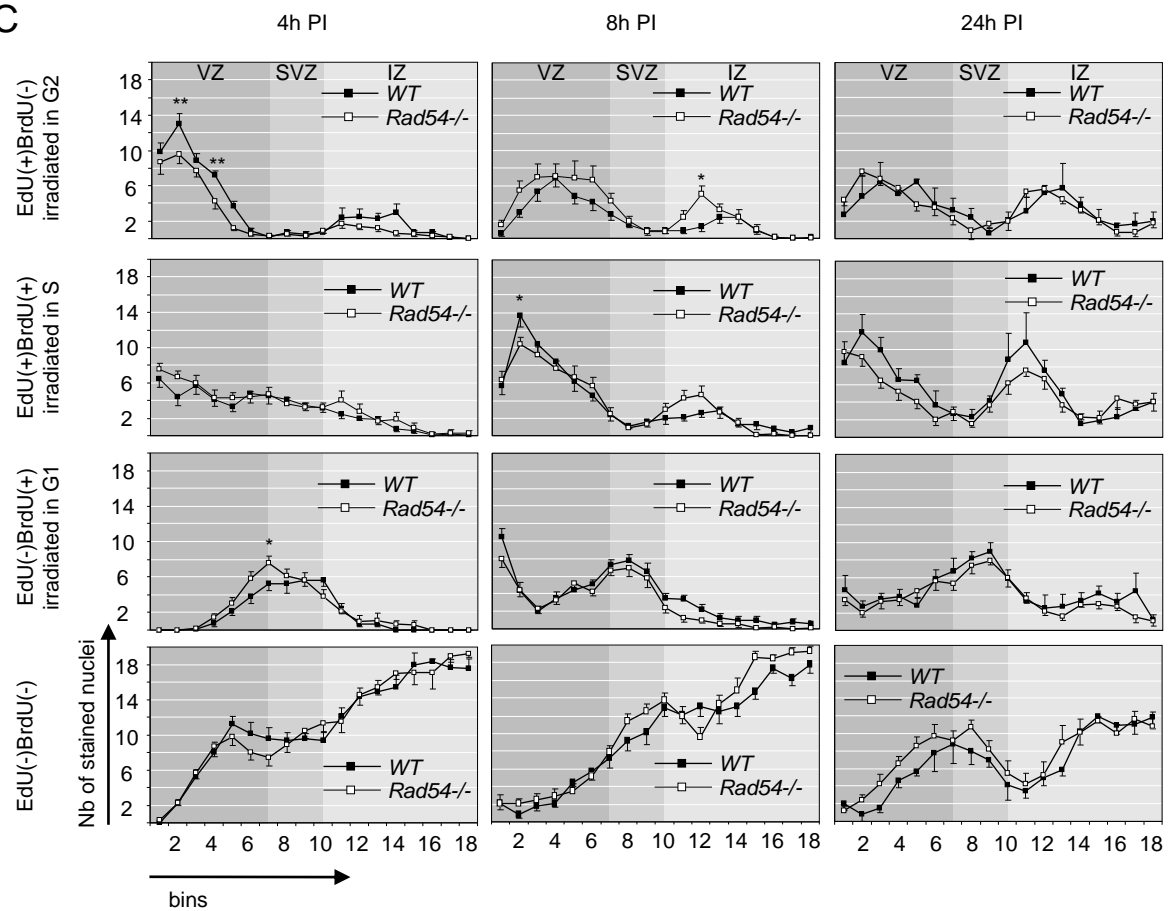

Fig.S3

Supplement: Figure S3 — Disruption of Rad54 has no effect on the progression of the cell cycle of NSPC in normal conditions. A Scheme of the three experimental designs with analyses at 4 h, 8 h, and 24 h after the first injection of BrdU. B Coronal sections of the cerebral hemisphere of WT (top) and Rad54−/− (bottom) embryos coming from the protocol described in (A) at 4 h (left) or 24 h (right) after first injection of BrdU from embryos. The sections were stained for EdU (green) and BrdU (red). Ventricles are on the left of each section. Scale bars, 10 µm. C Number per bin of, from top to bottom, EdU(+)BrdU(−), EdU(+)BrdU(+), EdU(−)BrdU(+) and EdU(−)BrdU(−) nuclei of embryos coming from the protocols described in (A) with analyses 4 h (left), 8 h (middle) and 24 h (right) after the first injection of BrdU. Mean values ± SEM were calculated from WT (plain squares) and Rad54−/− (open squares) embryos from at least three distinct litters for each genotype. All statistical analyses were performed as described in the Material and Methods. (PDF) [file pone.0037194.s003.pdf]
