# Supplementary material for: In vivo Importance of Homologous Recombination DNA Repair for Mouse Neural Stem and Progenitor Cells
Source: PLoS One. 2012 May 29;7(5):e37194. doi: 10.1371/journal.pone.0037194 (PMC3362579; doi:10.1371/journal.pone.0037194)
Supplement: Table S1 — Survival after birth of in utero irradiated embryos at E14.5. In each group, half were killed at 3.5 months, totally healthy. These others were alive up to 8 months. (DOC) [file pone.0037194.s004.doc]

**Supporting Table**

**Table S1** Survival after birth of *in utero* irradiated embryos at E14.5. In each group, half were killed at 3.5 months, totally healthy. These others were alive up to 8 months.

|  | 1 Gy | 2 Gy |
| --- | --- | --- |
| *WT* | 100 % (29/29, 5 litters) | 100 % (17/17, 4 litters) |
| *Rad54-/-* | 100 % (10/10, 2 litters) | 0 % (0 / 3 litters) |
